# Prediction of successful aging using ensemble machine learning algorithms

**DOI:** 10.1186/s12911-022-02001-6

**Published:** 2022-10-03

**Authors:** Zahra Asghari Varzaneh, Mostafa Shanbehzadeh, Hadi Kazemi-Arpanahi

**Affiliations:** 1 Department of Computer Science, Faculty of Mathematics and Computer, Shahid Bahonar University of Kerman, Kerman, Iran; 2 Department of Health Information Technology, School of Paramedical, Ilam University of Medical Sciences, Ilam, Iran; 3 Department of Health Information Technology, Abadan University of Medical Sciences, Abadan, Iran; 4 Student Research Committee, Abadan University of Medical Sciences, Abadan, Iran

**Keywords:** Machine learning, Ensemble learning, Quality of life, Aged

## Abstract

**Background:**

Aging is a chief risk factor for most chronic illnesses and infirmities. The growth in the aged population increases medical costs, thus imposing a heavy financial burden on families and communities. Successful aging (SA) is a positive and qualitative view of aging. From a biomedical perspective, SA is defined as the absence of diseases or disability disorders. This is distinct from normal aging, which is associated with age-related deterioration in physical and cognitive functions. From a social perspective, SA highlights life satisfaction and individual well-being, usually attained through socialization. It is an abstract and multidimensional concept surrounded by imprecision about its definition and measurement. Our study attempted to find the most effective features of SA as defined by Rowe and Kahn's theory. The determined features were used as input parameters of six machine learning (ML) algorithms to create and validate predictive models for SA.

**Methods:**

In this retrospective study, the raw data set was first pre-processed; then, based on the data of a sample of 983, five basic ML techniques including artificial neural network, decision tree, support vector machine, Naïve Bayes, and k-nearest neighbors (K-NN) with one ensemble method (that gathers 30 K-NN algorithms as weak learners) were trained. Finally, the prediction result was yielded using the majority vote method based on the output of the generated base models.

**Results:**

The experimental results revealed that the predictive system has been more successful in predicting SA with a 93% precision, 92.40% specificity, 87.80% sensitivity, 90.31% F-measure, 89.62% accuracy, and a ROC of 96.10%, using a five-fold cross-validation procedure.

**Conclusions:**

Our results showed that ML techniques potentially have satisfactory performance in supporting the SA-related decisions of social and health policymakers. The KNN-based ensemble algorithm is superior to the other ML models in classifying people into SA and non-SA classes.

## Background

According to the World Health Organization (WHO), the aging population is growing and will reach nearly 1.6 billion by 2050 (almost 16% of the entire world population) [1]. Any country where 7% of the population is over 60 years old is considered an aged country [2]. At present, we are witnessing dramatic socio-demographic and lifestyle changes, and especially in developed countries, we are facing a transition from an aged to a super-aged population [3–5]. Iran is no exception to this transition; due to the changes in epidemiological aspects of diseases, it is estimated to face a sudden increase in the elderly population in the next two decades [6].

Due to recent advances in medical and social science, human life expectancy has generally been increasing in both developed and developing countries. Accordingly, all countries are grappling with the phenomenon of aging [7, 8]. The increase in life expectancy is in itself an important achievement of science. However, the growth of the elderly population raises the costs of social welfare and the necessary care for the elderly [9]. In addition to changes in the demographic situation of society, the growth of the elderly population has altered the epidemiological trends of diseases and raised the rate of chronic diseases worldwide[10]. Aging has increased the economic burden on families and governments. As such, besides health and medical aspects, the phenomenon of aging has led to global socio-economic concerns [11, 12].

The elderly's quality of life (QOL) and how they spend their lives in this period are critical. Although longevity is universally desired, the improvement of QOL indicators and reduction of the disease burden in old age are more important to both individuals and society than the number of years added to human life [13].

Successful aging (SA) is a concept dealing with population aging issues. SA is a multidimensional and interdisciplinary concept because the aging process differs for each person [14, 15]. Although there is no formal definition of SA, there is general agreement that people with SA should be free from chronic diseases and have good physical and mental functions [16, 17]. An operative theory regarding SA has been proposed by Rowe and Kahn [18] and has three dimensions: active engagement with life, absence of disease or disability, and appropriate physical and cognitive functioning [19]. According to this theory, which is largely accepted by academic circles [20], SA is a qualitative description of aging that shows the elderly person's adaptation to the physical, spiritual, and social changes caused by the passage of time [21].

The emphasis of numerous studies on SA has changed from a single dimension (disease existence or functional deterioration) to the multidimensional idea of SA, which is consistent with the WHO's definition of health; according to this definition, health is considered as a state of complete physical, mental, social, and spiritual well-being [22]. However, due to the inherent ambiguity in the meaning of this complex and multidimensional phenomenon, its definition has proven to be a difficult task [23].

A notable point about aging is that it is not exclusively influenced by genes, but non-genetic factors also significantly affect the aging process [17]. Prior investigations have generally focused on factors affecting SA, but there are no longitudinal studies on SA [24, 25]. The factors affecting SA are codependent and multifaceted, and conventional statistical models are not appropriate for this concept [26]. Over the past few decades, machine learning (ML) algorithms have played a key role in solving complex, multidimensional, and nonlinear problems [27]. Hence, it is possible to create an intelligent model to predict the presence or absence of SA. Still, scholars are always seeking ways to augment these techniques. Ensemble learning is one of these methods that has been demonstrated to improve ML performance [28]. Therefore, five basic and one hybrid ensemble ML models were developed and tested for SA prediction. Our study aimed to develop SA prediction models based on sociodemographic, clinical, and lifestyle factors, which can be used for early prediction of SA and to explore important predictors affecting its further progress.

## Methods

### Study design and setting

This research is a retrospective study that included 1115 adults in a database of Abadan University of Medical Sciences, Abadan, Iran, from January 2016 to August 2021.

Developed countries consider the age of 65 years as the onset of old age because a person qualifies for a pension. However, the United Nations and the WHO recognize 60 years and older as elderly [29, 30]. In this study, according to the WHO, people aged 60 years and older are considered elderly. Therefore, individuals younger than 60 years and incomplete case records with missing more than 70% of the data were excluded from the study.

**Fig. 1:**
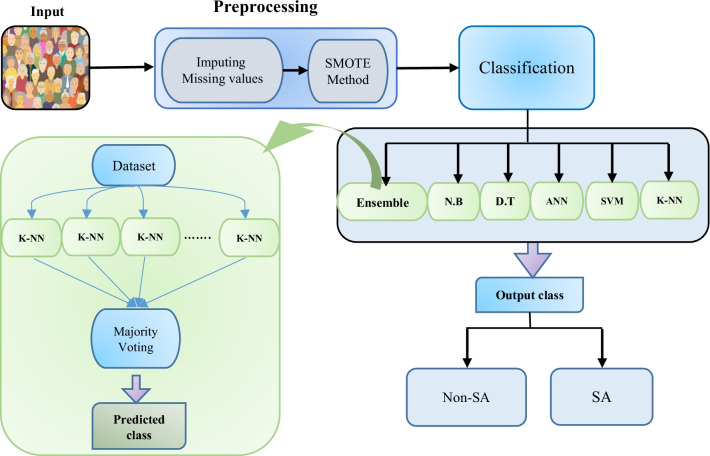
An overview of the proposed system

To predict whether a person has SA or non-SA status, five basic classification algorithms, including artificial neural network (ANN), decision tree (DT), support vector machine (SVM), Naïve Bayes (NB), and k-nearest neighbors (K-NN) models were first trained. Then, to promote the prediction accuracy of the models, a hybrid model called ensemble-based KNN was developed. This model gathers some weak learners as base classifiers. Each of these classifiers is trained, and ultimately, the prediction result is obtained using the majority vote method based on the output of the generated base models [31, 32]. Figure 1 displays an overview of the proposed system.

### Study variables

There is a large number of variables collected for the elderly in the EMR database. Thus, we checked the definition of the features included in the data dictionary section of the database to fully understand the data definitions and the choice of proper variables. The criteria for identifying the candidate variables related to SA were based on consultations with gerontologist experts and reviewing the relevant literature. Predictor and outcome variables are described as follows:

#### Sociodemographic factors

This class includes seven variables of age, sex, literacy level, marital status, occupation, income level, and insurance situation.

#### Clinical factors

This class includes hypertension, cardiovascular accident (CVA), bone disease, renal disease, liver disease, muscle disease, depression, convalescences, eye disease, diabetes, cancer, and other diseases.

The sociodemographic and clinical variables were extracted from aged adults’ electronic health records (EHR).

#### Behavioral and psychosocial factors

This class comprises the ability to perform activities of daily living (ADLs), life satisfaction, QOL, healthy lifestyle, social and interpersonal relationships, nutrition, physical activity, disease prevention activities, and tension (stress) management. These variables were defined as follows:

##### Ability to perform ADLs

This factor is measured by the Barthel Index, which has 10 questions to measure physical functioning. Barthel Index determines one's ability to perform basic ADLs, e.g., dressing, on a scale ranging from 0 to 100. Scores of 0–20 indicate severe dependence, 20–60 complete dependence, 61–90 moderate dependence, 91–99 partial dependence, and 100 complete independence [33]. In our study, an independent person is someone who has a score of 100 based on the Barthel index.

##### Life satisfaction

This variable was measured by the Life Satisfaction Scale developed by Diner et al. (1985). This scale consisted of 5 items measuring the cognitive component of well-being. Each statement has seven options and is scored from 1 to 7 (*strongly disagree* to *agree strongly*). The validity of this instrument was confirmed by Bayani et al. (2007) [34]. In this study, a person who is satisfied with life receives a score of > 20 on this scale.

##### QOL

To measure this variable, the 36-Item Short-Form Survey (SF-36) was administered. This self-report questionnaire consists of 36 items and eight domains: physical function, social function, physical role-playing, emotional role-playing, mental health, evaluations of vitality, physical pain, and general health. In addition to these sections, SF36 also provides two general measures of physical health (total physical component score (PCS)) and mental and social health (total mental component score (MCS)). The respondents' scores in each domain vary from 0 to 100, and a higher score means a better QOL. The validity and reliability of this questionnaire in the Iranian population have been confirmed [35–37]. Physical activity and social and interpersonal relationships are the SF-36 subcategories evaluated in the elderly. In addition, the overall score was calculated to measure the QOL of the elderly. In the present study, a score of 70 was considered as the cut-off point for this variable.

##### Healthy lifestyle

Lifestyle determination generally depends on the total score obtained and is calculated by obtaining a score of 42–98 indicating an unfavorable, 99–155 showing a medium, and 156–216 denoting a desirable lifestyle. It measures physical activity, exercise, recreation, healthy eating, stress management, and social and interpersonal relationships [38].

##### Nutrition status

The Mini Nutritional Assessment questionnaire was administered to measure the healthy nutritional status of the elderly. In this questionnaire, a score of 12 or greater indicates that the person is well-nourished and needs no further intervention. A score of 8–11 shows that the person is at risk of malnutrition. A score of 7 or less demonstrates that the person is malnourished. The cut-off point of this variable in our study is 12.

##### Stress management

The Stress-Management Questionnaire was used to describe the participant’s ability to cope with difficult and stressful situations. The total scores were divided into three levels of low (0–30), moderate (31–39), and high (40–50). The cut-off point of this variable in our study is 31.

##### Outcome variable

The outcome variable was categorized into SA (coded 1) or non-SA (coded 0) classes. In this study, SA was determined based on Raw and Khan's model which has three principal components: “absence of disease and disease-related disability”, “maintenance of high mental and physical function, “and “continued engagement with life” [39]. According to this model, the following inclusion criteria of SA were used: 1) absence of disease-related disability (the criteria met in this domain are being satisfied when adults have no disability and the number of chronic diseases ≤ 2), 2), maintenance of high mental and physical function (in this domain, the participants had a Mini-Mental State Examination for Dementia Screening (MMSE-DS) score of normal and a Bartle index of > 90), and 3) “continued engagement with life” (this domain was determined based on employment, participation in social activities, religious activities, volunteering activities, and lifelong learning. The participants had to have at least three out of these five criteria) [24, 40–43].

### Data preprocessing

When the collected raw data were processed by ML models, they did not have acceptable performance and the classifiers’ prediction accuracy was low. Therefore, data preprocessing methods were adopted to obtain the best models for SA identification. The dataset used in this paper contained missing values. Deleting these data from the data set would reduce the quality of the data because the data might contain useful information that could affect the prediction. There are several ways to solve the problem of missing value. We filled missing values with the mean value of the respective feature in the data set. Another problem with the collected data was unbalanced data. Unbalanced data class distribution occurs when the number of samples related to one class is significantly less than the number of samples belonging to another class. This deteriorates the efficiency of ML algorithms [44]. We used the synthetic minority oversampling technique (SMOTE) to deal with the problem of an unbalanced dataset [45, 46].

SMOTE selects a random sample from the minority class and determines k nearest neighbors for this sample. Then, a vector between the current sample and a chosen neighbor is determined. The synthetic instances are generated by multiplying this vector with a random number between 0 and 1. This action is similar to slightly moving a data point in the direction of its neighbor. Thus, the generated data point is not an exact copy of an existing data point and does not significantly differ from known observations in the minority class. We applied oversampling only to the training data; by so doing, none of the information in the validation data was being used to create synthetic observations. Therefore, these results should be generalizable. According to the tests performed, the validation results were consistent with the results of unseen test data. The data set contained 983 records before and 1430 records after data balancing.

### Feature selection

We used the feature selection method to reduce the dataset dimension and augment the ML performance. Feature selection in a high-dimensional dataset is one of the most important data mining steps, eliminating redundancy and unrelated features. Feature selection involves the use of statistical methods to reduce the dataset dimension. Briefly, some advantages of this process include improving the mining performance, preventing overfitting the algorithms, increasing the computational capability, accelerating the data mining process, and enhancing understandability [47–51]. In this study, the chi-square test was used to determines the important factors affecting the SA. *P* < 0.05 was considered statistically significant. After the correlation analysis, a univariate regression analysis was conducted to improve the accuracy and significant variables. This is one of the basic prerequisites of ML techniques; in this regard, the variables were entered into the model that had high prognostic power.

### Model development

#### Basic ML algorithms

We applied supervised learning techniques for SA prediction. Several basic algorithms such as KNN, SVM, DT, NB, and RF were used to classify whether people belong to the class of SA or non-SA.

##### ANN

An ANN is an ML algorithm inspired by the natural nervous system in processing information. The structure of the neural network consists of a large number of processing elements (neurons) that communicate with one another through weights. The neural network has a nonlinear mechanism that can process in parallel, learn, and make decisions. ANN modifies its weighted connections using a set of learning examples. The final effects of the learning process are the adjustment of the parameters of a network that can be retrained in new environmental conditions [52, 53].

##### DT

Decision trees and decision rules are efficient ways to solve classification problems. These techniques are supervised learning methods that construct decision trees using a set of input and output samples. A typical decision tree learning system implements a top-down strategy to find a solution in a portion of the search space. The main elements of the decision tree include decision nodes where the data are partitioned and leaves that represent the output. In the tree learning process, the feature that causes the greatest change in entropy is first selected, and the data set is divided based on this feature. The same process is then repeated for each of the created subsets and continues until the resulting subsets are of minimal purity [54, 55].

##### NB

This algorithm is a generalization of the Bayesian theorem in which the attributes are assumed to be independent of one another. NB is a probabilistic model, and the process it follows involves the calculation of the probability of a data sample belonging to a particular class [56, 57]. NB is sometimes called simple Bayes or independence Bayes. It is easy to create this algorithm, and it does not need to estimate complex initial parameters, i.e., it can be used for a large set of data and has high accuracy and speed when using large databases. However, the lack of access to data probabilities and conditional independence of classes are the problems of these algorithms [58, 59].

##### SVM

SVM is a supervised learning method used for classification and regression and is a linear classifier [60]. The purpose of the SVM is to find the best classifier to distinguish between samples of two classes in the training data. For linearly separable datasets, a linear function for a hyper-plan passing through the middle of two layers separates the two. Since there are many such linear hyper-planes, SVM ensures that the best function is found by maximizing the margin between the two classes. Intuitively, a margin is the amount of space or the separator of two classes defined by hyper-planes [61, 62].

##### KNN

KNN is an unsupervised classification algorithm. The KNN classifier finds a K group of objects in the training set that is closest to the test data. The three main elements in this procedure include a set of labeled objects, a distance criterion for calculating the distance between objects, and a value that determines the number of nearest neighbors. To classify an unlabeled object, the distance of this object from the labeled objects is calculated; then, k nearest neighbors of the object are identified and their class labels are used to determine the class label of the unlabeled object [63, 64].

### Ensemble learning algorithm

Ensemble learning models are ML methods in which several weak learners, which are base models, are trained to solve a problem and combined to achieve better results [65]. When weak models are properly combined, they can produce more accurate or stable models [23, 24]. In ML models, the choice of algorithms is crucial to obtaining good results. Model selection depends on many variables in the problem such as data amount, data dimensions, and distribution hypothesis [31, 66]. In many cases, especially bagging and boosting methods, a single base learning algorithm is utilized. Consequently, there are several similar basic models trained in different ways, which are called homogeneous ensemble models [32]. In other methods, such as the stacking method, different types of basic learning algorithms are used, which are called heterogeneous ensemble models [67, 68].

The proposed hybrid algorithm consists of two main steps. In the first step, preprocessing was performed on the dataset, as described in the previous section. In the second step, classification algorithms are applied to the dataset, and the results of the classification evaluation are compared. The classifier proposed in this paper is based on the ensemble learning algorithm in which the KNN algorithm is utilized as the base learner.

The ensemble learning model tries to produce a model for data classification that combines several learners such that they have high performance compared to the main learners [69, 70]. A key ensemble learning method is the bagging method, in which each classifier views a subset of the original data and builds its model based on it. The selection of this subset is performed with replacement from the full dataset [70, 71].

The proposed model is similar to the bagging method. In this model, 30 weak learners are used as basic learners. The weak learners used in this paper are KNN. The choice of this algorithm is motivated by its simplicity of implementation and stability to variations in the training dataset. The subset of features used in this step includes 21 of the best data features, obtained by the chi-squared test. These parameters determine the correlation between the input variables and the output class.

Based on the experimental results, the use of conventional feature selection methods such as relief and minimum redundancy maximum relevance (mRMR) on the tested data had a negative impact on the performance of data mining algorithms; consequently, we decided to adopt the random feature selection method in the proposed method. For each learner, a subset of input data features is selected randomly and with replacement. Subsequently, a subset of the training sample is trained by the KNN algorithm based on these features.

Each time a random subspace is selected, a new set of k nearest neighbors is computed. Therefore, the output of the models will be different. After training the learners, the learning algorithms (KNNs) are collected for the majority of votes regarding the class membership of the test sample according to the selected feature subset. The weight of all learners is considered equal, i.e., in the voting phase, all the algorithms have the same chance. Thus, the proposed model uses a combination of two bagging techniques and feature selection methods and attempts to classify datasets with the highest efficiency by selecting the best data features.

### Evaluation of the ML models

**Fig. 2:**
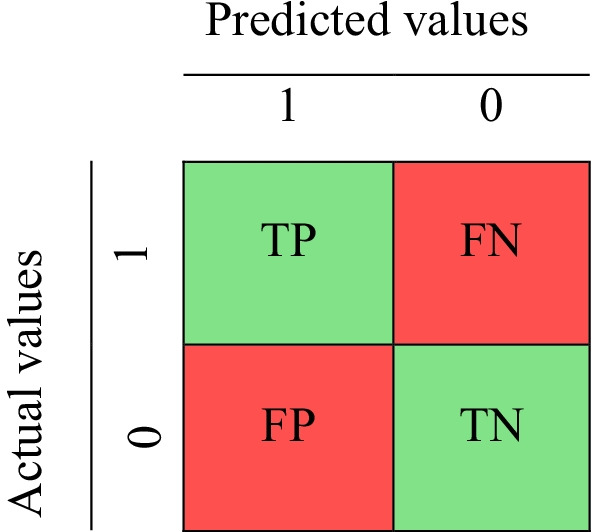
Structure of the confusion matrix

The performance of the proposed models on the selected data set was evaluated. All the experiments were performed in MATLAB 2019. The evaluation criteria included accuracy, precision, sensitivity, specificity, and F-measure [27]. A confusion matrix (Fig. 2) was used to calculate the value of the evaluation criteria [72]. Each column of the matrix indicates an instance of the predicted value, and each row contains a real (correct) instance.

The calculation formula for each criterion is defined in Table 1. Another important evaluation criterion used in this paper is the area under the curve of the receiver operating characteristics curve (AUC-ROC), which determines the extent to which the model can differentiate between classes [73]. Furthermore, the fivefold cross-validation method was adopted to evaluate the performance of the algorithms.

**Table 1:** Definition of evaluation criteria

Performance metrics	Definitions
Precision	TP/(TP + FP)
Specificity /true negative rate (TNR)	TN/(TN + FP)
Sensitivity/true positive rate (TPR) or recall	TP/(TP + FN)
Accuracy	(TP + TN)/(TP + TN + FP + FN)
F-measure	(2 × precision × recall)/(precision + recall)

^*^True Positive (TP), True Negative (TN), False Positive (FP), False Negative (FN)

Hyperparameters in ML are parameters determined when configuring the model to control the learning process. These hyper-parameters are used to improve model learning, and their values are set before starting the model learning process. Not all hyper-parameters are equally important. Some hyper-parameters have a greater impact on the performance of the ML algorithm. In this paper, we value some of the most important hyperparameters for the ML models given in Table 2.

**Table 2:** Hyperparameters for ML algorithms

ML Algorithms	Hyperparameters
DT	min_samples_split = 2, min_samples_leaf = 1, max_features = none
SVM	C = 1, kernel = rbf, gamma = scale
NB	Alpha = 1.0,
ANN	Learning rate = 0.01, Number of Hidden Units = 2
KNN	n_neighbors (K) = 5

## Results

### Participants' characteristics

After reviewing the data set, 60 and 40 records of respectively non-SA and SA that had more than 70% missing values were removed. Of the remaining records, 32 records belonging to people less than 60 years old were discarded, and 983 records were finalized (239 SA and 744 non-SA). Of 983 individuals in this retrospective study, 561 (57.07%) were male and 422 (42.93%) were women, and the participants' median age was 77.25 (interquartile 60–103). The results of the chi-square test to determine the most important factors associated with SA are listed in Table 3. Variables entering univariant regression that have a p-value of less than 0.05 as presented in Table 3.

**Table 3:** Results of correlation of factors affecting SA

No.	Class	Variable name	Variable type	Concept codes	Frequency	chi-square	*P*_-Value_
1	Socio-demographic	Age (years)	Polynomial	1 (65–69) 2 (70–79) 3 (> 80)	1 (413) 2 (283) 3 (287)	0.073	0.036
2		Sex	Binominal	1 (Female) 2 (Male)	1 (422) 2 (561)	0.158	< 0.01
3		Educational level	Polynomial	1 (No literacy) 2 (Elementary) 3 (Diploma) 4 (Academic)	1 (154) 2 (297) 3 (445) 4 (87)	0.60	0.206
4		Marital status	Polynomial	1 (married) 2 (single) 3 (divorced) 4 (widowed)	1 (574) 2 (89) 3 (18) 4 (302)	0.036	0.651
5		Occupation	Polynomial	1 (no job) 2 (housekeeper) 3 (retired) 4 (self-employment)	1 (112) 2 (324) 3 (443) 4 (104)	0.068	0.117
6		Income level	Binominal	1 (under the poverty line) 2 (on poverty line)	1 (521) 2 (426)	0.138	< 0.01
7		Insurance status	Binominal	1 (Have) 2 (Haven’t)	1 (623) 2 (360)	0.005	0.853
8	Disease comorbidities	Hypertension	Binomial	1 (Yes) 2 (No)	1 (714) 2 (269)	0.275	< 0.01
9		Cardiovascular accident (CVA)	Binomial	1 (Yes) 2 (No)	1 (268) 2 (715)	0.249	< 0.01
10		Bone disease	Binomial	1 (Yes) 2 (No)	1 (399) 2 (584)	0.070	0.013
11		Renal disease	Binomial	1 (Yes) 2 (No)	1 (264) 2 (719)	0.027	0.344
12		Liver disease	Binomial	1 (Yes) 2 (No)	1 (120) 2 (863)	0.054	0.057
13		Muscle disease	Binomial	1 (Yes) 2 (No)	1 (335) 2 (648)	0.116	< 0.01
14		Depression	Binomial	1 (Yes) 2 (No)	1 (388) 2 (595)	0.74	< 0.01
15		Convalescences	Binomial	1 (Yes) 2 (No)	1 (170) 2 (813)	0.058	0.038
16		Eye disease	Binomial	1 (Yes) 2 (No)	1 (460) 2 (523)	0.017	0.540
17		Diabetes	Binomial	1 (Yes) 2 (No)	1 (722) 2 (743)	0.264	< 0.01
18		Cancer	Binomial	1 (Yes) 2 (No)	1 (88) 2 (895)	0.107	< 0.01
19		Other diseases	Binomial	1 (Yes) 2 (No)	1 (456) 2 (527)	0.025	0.678
20	Behavioral and psychosocial factors	Tension management	Binomial	1 (Yes) 2 (No)	1 (560) 2 (423)	0.063	0.025
21		Social and interpersonal relationships	Binomial	1 (Weak) 2 (Strong)	9 (399) 10 (584)	0.062	0.028
22		Life satisfaction	Binomial	1 (Pleasant) 2 (Unpleasant)	1 (466) 2 (517)	0.138	< 0.01
23		Healthy lifestyle	Polynomial	1 (Low) 2 (Medium) 3 (High)	1 (377) 2 (538) 3 (68)	0.075	0.029
24		Nutritional status	Binomial	1 (Bad) 2 (Good)	1 (351) 2 (632)	0.132	< 0.01
25		Ability to perform daily activities	Binomial	1 (dependence) 2 (independence)	1 (471) 2 (512)	0.149	< 0.01
26		Quality of life	Binomial	3 (Low) 4 (High)	1 (465) 2 (518)	0.205	< 0.01
27		Physical activity	Binomial	1 (Low) 2 (High)	1 (192) 2 (791)	0.162	< 0.01
28		Disease prevention activities	Binomial	1 (Have) 2 (Haven't)	1 (472) 2 (511)	0.070	0.013

Table 4 demonstrates the results of univariant regression. Significant variables extracted from univariate regression were entered into the ML algorithms.

**Table 4:** Results of univariant regression

No.	Class	Variable name	Concept codes	Odd ratio (OR)	CI (confidence interval)	*P*–Value
1	Socio–demographic	Age (years)	1 (65–69)	1.000	–	–
			2 (70–79)	0.658	0.236–0.785	< 0.001
			3 (> 80)	0.358	0.125–0.527	0.006
2		Sex	1 (Female)	1.000	–	–
			2 (Male)	0.435	0.217–0.784	< 0.001
3		Income level	1 (under the poverty line)	1.000	–	–
			2 (on poverty line)	1.365	1.215–1.606	0.003
4		Insurance status	1 (Have)	1.000	–	–
			2 (Haven’t)	0.635	0.487–0.963	0.013
5	Disease comorbidities	Hypertension	1 (Yes)	1.000	–	–
			2 (No)	0.321	0.221–0.425	< 0.001
6		Cardiovascular accident (CVA)	1 (Yes)	1.000	–	–
			2 (No)	1.369	1.023–1.409	0.005
7		Bone disease	1 (Yes)	1.000	–	–
			2 (No)	1.458	1.369–1.548	< 0.001
8		Muscle disease	1 (Yes)	1.000	–	–
			2 (No)	1.745	1.678–1.978	< 0.001
9		Depression	1 (Yes)	1.000	–	–
			2 (No)	2.360	2.023–2.415	0.018
10		Convalescences	1 (Yes)	1.000	–	–
			2 (No)	0.368	0.234–0.478	< 0.001
11		Diabetes	1 (Yes)	1.000	–	–
			2 (No)	1.369	1.023–1.409	0.005
12		Cancer	1 (Yes)	1.000	–	–
			2 (No)	2.364	1.998–2.419	< 0.001
13	Behavioral and psychosocial factors	Tension management	1 (Yes)	1.000	–	–
			2 (No)	0.425	0.365–0.574	< 0.001
14		Social and interpersonal relationships	1 (Weak)	1.000	–	–
			2 (Strong)	1.785	1.561–1.941	< 0.001
15		Life satisfaction	1 (Pleasant)	1.000	–	–
			2 (Unpleasant)	0.369	0.236–0.578	< 0.001
16		Healthy lifestyle	1 (Low)	1	–	–
			2 (Medium)	1.236	1.010–1.368	0.029
			3 (High)	2.360	2.263–2.684	0.001
17		Nutritional status	1 (Bad)	1.000	–	–
			2 (Good)	1.466	1.231–1.785	< 0.001
18		Ability to perform daily activities	1 (dependence)	1.000	–	–
			2 (independence)	2.365	2.020–2.474	< 0.001
19		Quality of life	3 (Low)	1.000	–	–
			4 (High)	1.386	1.120–1.585	< 0.001
20		Physical activity	1 (Low)	1.000	–	–
			2 (High)	1.365	1.120–1.485	< 0.001
21		Disease prevention activities	1 (Have)	1.000	–	–
			2 (Haven't)	0.368	0.234–0.478	< 0.001

### Selected features

Based on Table 4, the determinant factors of age (years), sex, income level, insurance situation, hypertension, CVA, bone disease, muscle disease, depression, convalescences, diabetes, cancer, stress management, social and interpersonal relationships, life satisfaction, healthy lifestyle, nutrition status, the ability to perform ADLs, QOL, physical activity, and disease prevention activities correlated with the output class at *P* < 0.05. Therefore, these factors were considered the most critical factors determining SA in aged persons. The seven variables of educational level, marital status, occupation, renal, liver, eye, and other diseases (with *P* > 0.05) did not show any significant correlation with the output class and were excluded from the data mining process. The 21 features were entered into univariant regression. Based on Table 4, 21 features significant in SA were used as inputs to develop basic ML models.

### Performance of the ML models

Table 5 shows the performance results of ML models. The precision criterion calculates the ratio of the number of people whose classifier has placed them in a positive class (SA) and are positive. According to this criterion, the ANN algorithm is the weakest algorithm and the proposed KNN-based ensemble algorithm has the greatest performance. The precision value in this algorithm is 93%. The SVM algorithm has the best value in terms of the recall criteria. This value is equal to 97.5% for the SVM algorithm and, therefore, it has the highest performance compared to other algorithms in identifying all people with SA. The KNN-based ensemble algorithm is better than other ML models in identifying all people who do not have SA. This means that this algorithm is the most successful in terms of specificity with a value of 92.4%. It is known that an algorithm is successful when it can establish a good balance between the two values of recall and specificity. The algorithm proposed in this paper has established the best balance between these two criteria. The criterion that considers both recall and specificity parameters is called the F-measure, whose value in the KNN-based ensemble algorithm is 90.3% and higher than the other compared models.

**Table 5:** The result of evaluating the efficiency of ML models

Model	Precision	Recall	Specificity	F-measure	Accuracy	AUC
DT	74	73.2	73.7	73.6	73.5	80
95% CI	(0.72, 0.752)	(0.725,0.74)	(0.719,0.75.2)	(0.71,0.747)	(0.71,0.759)	(0.79, 0.813)
Stanandard deviation (SD)	0.041	0.013	0.029	0.038	0.0217	0.029
SVM	78	97.5	81.6	86.3	85.1	95
95% CI	(0.763, 0.791)	(0.763,0.78)	(0.792,0.83)	(0.839,0.884)	(0.845,0.87)	(0.937, 0.961)
SD	0.035	0.019	0.035	0.102	0.042	0.018
NB	65	70.6	67.6	67.6	68.6	74
95% CI	(0.631, 0.67)	(0.69,0.713)	(0.659,0.692)	(0.658,0.685)	(0.669,0.69)	(0.725, 0.763)
SD	0.024	0.022	0.030	0.0173	0.015	0.037
ANN	48	64.7	77.2	89.3	77.1	78.2
95% CI	(0.462, 0.499)	(0.615,0.67)	(0.764,0.788)	(0.883,0.907)	(0.759,0.78)	(0.761, 0.793)
SD	0.06	0.057	0.035	0.041	0.012	0.02
KNN	90	72.1	86.6	80	73.3	91
95% CI	(0.886, 0.914)	(0.715,0.73)	(0.852,0.887)	(0.780,0.817)	(0.715,0.74)	(0.89, 0.925)
SD	0.048	0.019	0.051	0.0227	0.018	0.012
Ensemble 1 (KNN)	93	87.8	92.4	90.3	89.6	96
95% CI	(0.917, 0.941)	(0.86,0.893)	(0.919,0.941)	(0.89,0.917)	(0.874,0.91)	(0.951, 0.973)
SD	0.03	0.024	0.0107	0.0162	0.052	0.027
Ensemble 2 (Bag Tree)	82	86.3	82.8	85.8	84.4	90
95% CI	(0.802, 0.841)	(0.851,0.87)	(0.812,0.845)	(0.832,0.871)	(0.83,0.861)	(0.891, 0.817)
SD	0.03	0.012	0.031	0.026	0.039	0.032

**Fig. 3:**
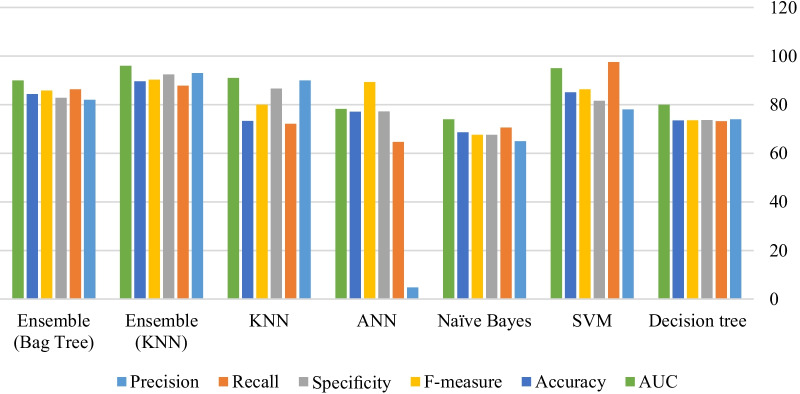
An overview of the comparison of ML algorithms

The most basic and simplest measure of the quality of a classifier is accuracy, which generally shows its quality in the correct detection of samples. The KNN-based ensemble algorithm is the best classifier with a value of 89.6%, and the NB algorithm has the lowest classification accuracy. The algorithm presented in this paper has the best performance in terms of the AUC criteria, i. e. the AUC-ROC curve in this algorithm is more than that of the other classifiers. Figure 3 depicts a bar chart to compare machine learning algorithms in terms of accuracy, precision, sensitivity, (recall), specificity, F-measure, and AUC.

**Fig. 4:**
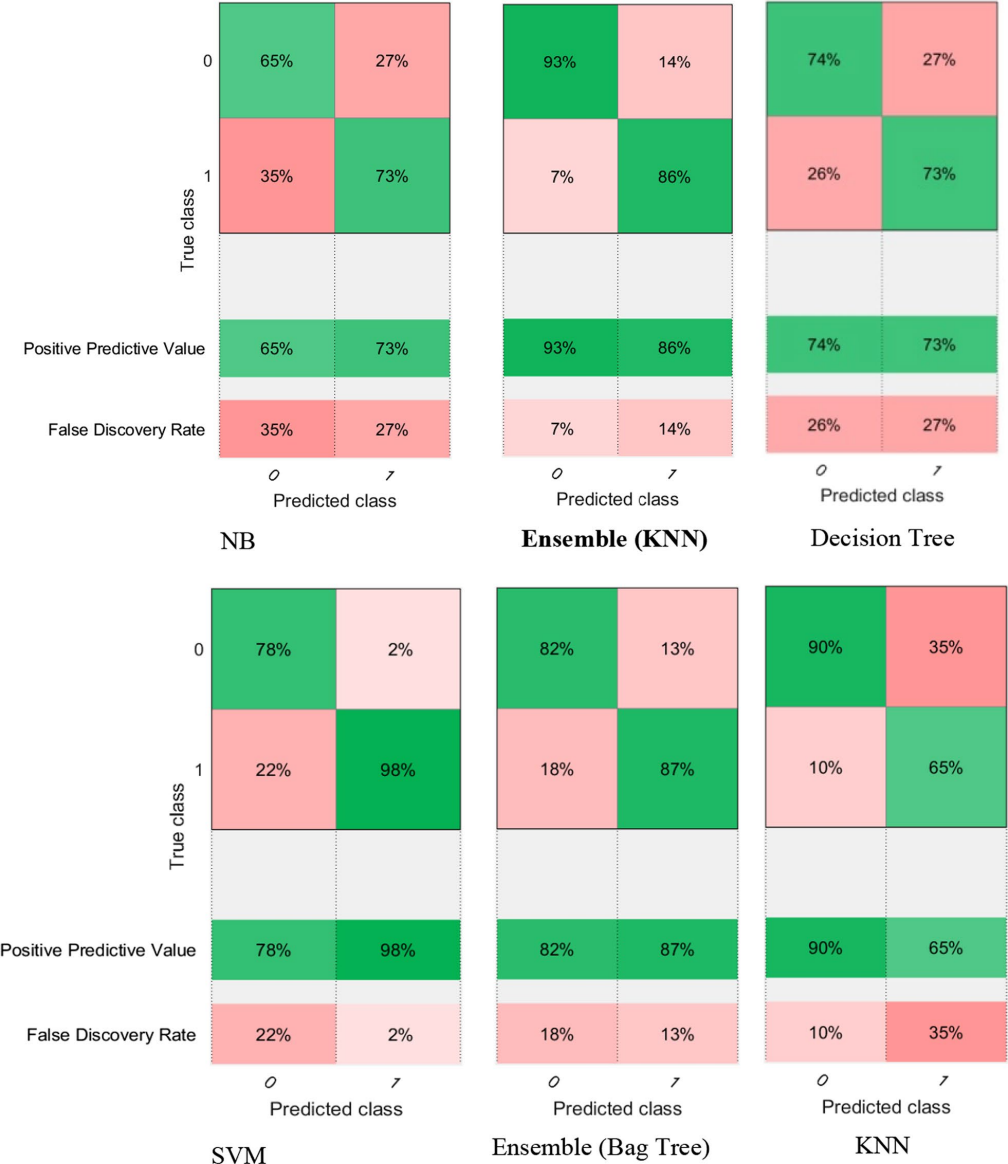
Confusion matrix of classifiers (0 indicates a SA class and 1 indicates none-SA class.)

**Fig. 5:**
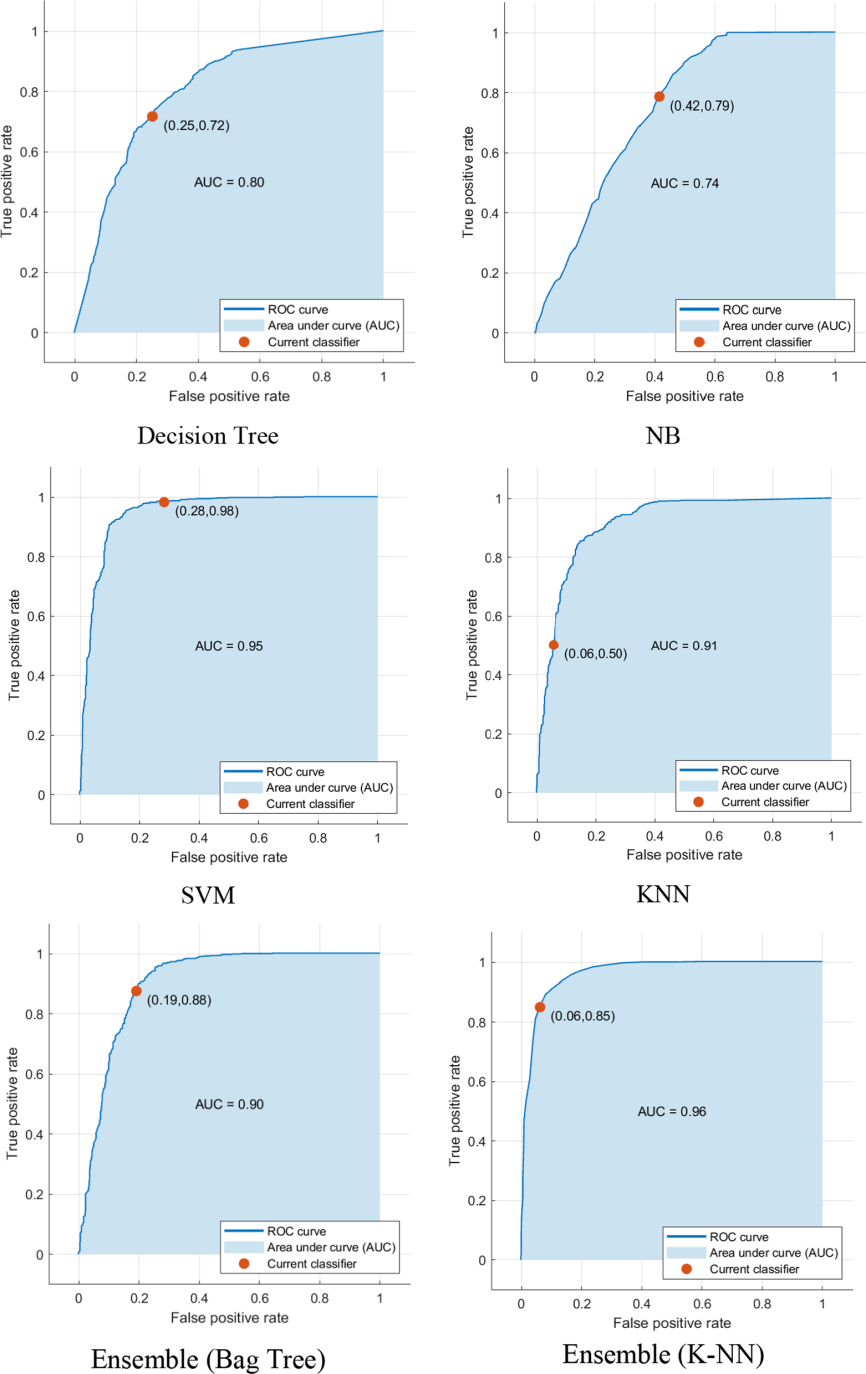
ROC for ML algorithms

Figure 4 illustrates the confusion matrix of all the classifiers, summarizing the performance of each classification model. These tables show the results of the classification based on the actual information available. The dimensions of these matrices are 2 * 2, i.e., the number of classes of data is 2, and each sample can be in one of two classes: 0 (unsuccessful aging) or 1 (SA). Based on the values calculated in the confusion matrix, different criteria for classification evaluation and accuracy measurement can be defined. By looking at these matrices, it can be concluded that the performance of the KNN-based ensemble algorithm is higher than the other classifiers. The ROC curve of all classifiers is depicted in Fig. 5. According to this figure, the proposed algorithm outperforms the other algorithms, which is confirmed by the numerical value obtained for the AUC parameter.

## Discussion

According to Rowe and Kahn’s theory, we measured SA in terms of three dimensions of physiological, cognitive-psychological, and social function [18]. We intended to develop prediction models that take clinical and lifestyle variables as inputs and predict whether the individual has SA or not. Our findings provide significant insights into SA likelihood assessment. In line with our primary assumption, the developed ML approach yielded a strong classifier of SA status. Herein, we presented a new method that applies five basic MLs and a hybrid ensemble technique to predict SA.

Numerous ML techniques can be used to develop a prediction model. The existing ML techniques have numerous basic model assumptions, preventing their successful implementation. When the dataset is highly varied and noisy, as is the case for the SA data that are naturally multidimensional and heterogeneous, it is not clear which technique is appropriate because it is usually difficult to validate basic assumptions. Besides, no single ML technique provides acceptable prediction results. Researchers and scientists are always looking for trained ML models that have accurate and stable performance. In practice, however, the results of training models are not perfect, as sometimes only a few prejudiced models can be attained. By calculating the chance of occurrence of each outcome, if there are some independent models, the performance of the hybrid model is much better than a single model. Indeed, ensemble learning combines several models with moderate performance to achieve a better-performing prediction model [74].

To the best of our knowledge, this was the first effort that applied ensemble ML classifiers for SA prediction. Nevertheless, some studies have been conducted on the application of the ensemble method to predict and identify other social aspects of aging. For example, Paul applied ensemble ML classification algorithms to recognize daily living abilities among the elderly with HIV. After implementation, the gradient boosting algorithm gained an average AUC of 83% [75]. In the study by Zhou, several ML classifiers such as DT, gradient boosting decision tree (GBDT), Ada boosting, bagging, and RF were compared to classify the healthy behaviors of the elderly. They concluded that ensemble learning classifiers improved modeling performance [76].

Liaqat compared the performance of multiple ML and deep learning classifiers for multiple activity recognition in elderly people. The developed ensemble algorithm outperformed other algorithms with an accuracy of 98% [77]. The experimental results of two separate studies conducted by Byeon showed that the predictive performance of ensemble classifiers was the best for predicting mental and physical impairments of the aged living alone, with an accuracy of 87.4% and 0.67%, respectively [78]. Shen showed that ensemble techniques perform better than single models in identifying the clinical support requirements of the elderly [79].

Lee compared the performance of base-level and hybrid-level learners (ensemble methods) to predict depression among the elderly. The results showed that ensemble models improve modeling capabilities [80]. Lin et al. also compared the predictive performance of the bagging ensemble ML algorithm with other basic models such as linear regression, SVM, multilayer feedforward neural networks, and random forests to predict the functional outcomes of schizophrenia. Eventually, the bagging ensemble algorithm outperformed the other algorithms [12].

In this study, we presented the KNN-based ensemble to predict whether the tested people belong to the class of SA or non-SA. This method was a hybrid ML model. In the first step, the data were preprocessed to be suitable for use in data mining analysis. Then a KNN-based ensemble was presented which has a greater ability to predict the SA than basic ML models such as ANN, SVM, NB, DT, and KNN. The experimental results revealed that the predictive system has been more successful in predicting SA with a 93% precision, 92.40% specificity, 87.80% sensitivity, 90.31% F‑measure, 89.62% accuracy, and a ROC of 96.10% using a fivefold cross-validation procedure.

Our results showed a satisfactory level using ML to predict SA in an elderly population. The calculated metrics disclosed that measurements of the trained ML techniques based on the selected features accurately predicted SA. Improved forecast performance might be associated with feature selection using former theoretic and experiential studies, which can help to successfully decrease the number of unrelated or redundant variables in the model. Furthermore, to avoid overfitting, the technique of fivefold cross-validation was applied, which will also be helpful for the application of the model.

The novelty of our study lies in that we used an ensemble learning method. Our study highlights the power of ensemble ML vs. base ML techniques and how merging the power of several mixed ML algorithms can provide a more reliable accuracy, without bias. Although the ensemble ML in our study enhanced the prediction model performance, the predictive performance may be further enhanced by selecting other practical prediction models.

While our study offered an optimal performance in estimating the SA in aged people, it had some possible limitations that should be pointed out. First, this study retrospectively analyzed a dataset from a single database that influences the quality, comprehensiveness, and generalizability of data. By using this dataset, some inconsistent, inadequate, erroneous, and irregular data items could have undesirably impacted the prediction models. Thus, in the preprocessing phase, to improve data uniformity, the standard choice of each variable was determined based on the views of two gerontologists. Then, all the values outside the defined range (noisy fields) were specified and completed by a discussion with a gerontologist. Furthermore, the cases with more than 70% of blank fields were excluded and substituted by mean and mode values for constant and discrete variables separately. Second, this study only used six ML algorithms on a small sample dataset. The accuracy and generalizability of our models will increase if more ML techniques are tested on larger, multicenter, and prospective datasets. Third, an external validation method should be adopted to confirm the results of the present study. Fourth, this study did not investigate a causal relationship between the predictor and the outcome variables. Although this was not the main goal of this research, future studies are advised to determine a set of longitudinal factors associated with SA. Fifth, part of the data was collected during the coronavirus disease 2019 (COVID-19) pandemic, which may have affected the health of elderly people. This pandemic may influence the elderly's QOL, mental health, and the development of chronic complications. In this research, QOL and stress management questionnaires were used to measure the impact of the pandemic on SA. People who died due to COVID-19 were excluded from the study, and for those who suffered from mobility, their ability to perform ADLs was evaluated by Barthel's index. Furthermore, the stress management questionnaire measures the level of coping with environmental stress and removes the effect of COVID-19 as a confounding factor. In addition, the QOL questionnaire measures both physical and mental health, which neutralizes the physical and mental effects of COVID-19 as a confounding factor.

## Conclusions

The main goal of this study was to evaluate several ML models (basic vs. ensemble) to predict SA. The findings revealed that the ensemble ML model is a promising approach for improving the prediction of SA. The present study may assist geriatricians and senior nurses in providing optimal supportive services and customized care for the elderly. Our developed prediction models also have the potential to provide healthcare managers and policymakers with a reliable and responsive tool to improve elderly outcomes. These predictive models may help promote SA probability. In future works, this model is expected to be applied and customized to other social problems.

## Data Availability

The datasets used and/or analyzed during the current study are available from the corresponding author on reasonable request. The design and performance of our study are described and justified in a research protocol. The protocol includes information regarding funding, sponsors (funded), institutional affiliations, potential conflicts of interest, incentives for subjects, and information regarding provisions for treating and/or compensating subjects who are harmed as a consequence of participation in the research study. This protocol is as follows:

## Abbreviations

SA: Successful aging
ML: Machine learning
ANN: Artificial neural network
DT: Decision tree
SVM: Support vector machine
NB: Naïve Bayes
KNN: K-nearest neighbors
WHO: World health organization
QOL: Quality of life
AUC-ROC: Area under the curve-receiver operating characteristics curve
TP: True positive
TN: True negative
FP: False positive
FN: False negative
GBD: Gradient boosting
GBDT: Gradient boosting decision tree
CI: Confidence interval
SD: Standard deviation
